# Habitat fragmentation and logging affect the occurrence of lesser mouse‐deer in tropical forest reserves

**DOI:** 10.1002/ece3.8745

**Published:** 2022-03-18

**Authors:** Muhammad Hazwan, Liza D. Samantha, Sze Ling Tee, Norizah Kamarudin, Ahmad R. Norhisham, Alex M. Lechner, Badrul Azhar

**Affiliations:** ^1^ Department of Forest Science and Biodiversity Faculty of Forestry and Environment Universiti Putra Malaysia Selangor Malaysia; ^2^ School of Environmental and Geographical Sciences University of Nottingham Malaysia Selangor Malaysia; ^3^ Lincoln Centre for Water and Planetary Health Lincoln UK; ^4^ School of Geography University of Lincoln Lincoln UK; ^5^ Biodiversity Unit Institute of Bioscience Universiti Putra Malaysia Selangor Malaysia

**Keywords:** camera trap, conservation, lowland dipterocarp, peat swamp

## Abstract

Due to rapid urbanization, logging, and agricultural expansion, forest fragmentation is negatively affecting native wildlife populations throughout the tropics. This study examined the effects of landscape and habitat characteristics on the lesser mouse‐deer, *Tragulus kanchil*, populations in Peninsular Malaysia. We conducted camera‐trap survey at 315 sampling points located within 8 forest reserves. An assessment of site‐level and landscape variables was conducted at each sampling point. Our study provides critical ecological information for managing and conserving understudied populations of *T. kanchil*. We found that the detection of *T. kanchil* was attributed to forest fragmentation in which forest patches had four times greater detection of *T. kanchil* than continuous forest. The detection of *T. kanchil* was nearly three times higher in peat swamp forest compared to lowland dipterocarp forests. Surprisingly, the detection of *T. kanchil* was higher in logged forests (logging ceased at least 30 years ago) than unlogged forests. The detection of *T. kanchil* increased with the presence of trees, particularly those with DBH of 5 cm to 45 cm, canopy cover, number of saplings and palms, number of dead fallen trees, and distance from nearest roads. However, detection decreased with a greater number of trees with DBH greater than 45 cm and higher elevations, and greater detections where creeping bamboo was abundant. We recommend that conservation stakeholders take the necessary steps (e.g., eradicating poaching, habitat degradation, and further deforestation) to support the conservation of mouse‐deer species and its natural habitats.

## Graphical abstract

Forest fragmentation adversely affects native wildlife populations throughout the tropics. This study provides critical ecological information for managing and conserving understudied populations of the lesser mouse‐deer. Conservation stakeholders should take the necessary steps to support the conservation of mouse‐deer species and its natural habitats.

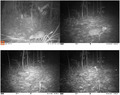

## 1 INTRODUCTION

Habitat fragmentation caused by human activities (e.g., road and railway, logging, agricultural expansion) is one of the major threats to global biodiversity as it leads to declines in nearly all taxonomic groups including birds, mammals, reptiles, amphibians, invertebrates, and plants (Aide et al., 2013; Fischer & Lindenmayer, 2007; Foley et al., 2007; Gibbons et al., 2000; Hobbs & Yates, 2003; Jamhuri et al., 2018; Laurance & Arrea, 2017; Sala et al., 2000; Samantha et al., 2020; Stuart et al., 2004; Tee et al., 2018). Habitat fragmentation is associated with increasing poaching and logging, and it adversely affects behavioral patterns of animal species, reproduction, and survival of animals (Azlan, 2006; Chaves et al., 2019; Laurance & Arrea, 2017; Ngoprasert et al., 2007).

Across its range, wild populations of chevrotain or mouse‐deer are declining because of habitat fragmentation, habitat destruction for timber extraction, and poaching (Adila et al., 2017; Heydon & Bulloh, 1997; Jamhuri et al., 2018; Nguyen et al., 2019; Tee et al., 2018). Chevrotain species inhabit primary and secondary lowland rainforests and, in all parts of their range, are hunted for food (Azhar et al., 2014; Luskin et al., 2014). The lesser mouse‐deer (*Tragulus kanchil*) is one of the smallest ungulate species on earth, and it is found in tropical forests across Southeast Asia (Matsubayashi et al., 2003; Ronald, 1991).

Mouse‐deer such as *T. kanchil* plays an important ecological role in forest ecosystems as they are prey for small and large carnivores and are seed dispersers (Feer, 1995; Kawanishi & Sunquist, 2008; Prasad & Sukumar, 2010; Ramesh et al., 2013). *Tragulus kanchil* consumes high energy food resources such as fallen fruits and also browses vegetation at the understory level, including leaves, tubers, and shoots (Bodmer, 1990; Prasad et al., 2010; Ramesh et al., 2013). Although *T. kanchil* is listed as the least‐concern species by the International Union for Conservation of Nature (IUCN), little has been published regarding its behavior and ecology in fragmented forest landscapes. In addition, Heydon and Bulloh (1997) showed that selective logging had a negative impact on mouse‐deer populations in Sabah. However, it is not yet known whether this would apply in other regions of Southeast Asia.

In this study, we examined the relationship between the occurrence (based on the number of animal detections) of *T. kanchil* and a range of environmental drivers, including habitat quality, and landscape and forest characteristics using nonintrusive motion‐triggered camera traps. The response of *T. kanchil* to forest fragmentation and habitat modification through logging is poorly understood due to their cryptic behavior. Our study aimed to provide vital information on *T. kanchil* ecology for forest wildlife management and conservation in the tropics, particularly fragmented forest landscapes.

## 2 METHODS

### 2.1 Study area

**FIGURE 1:**
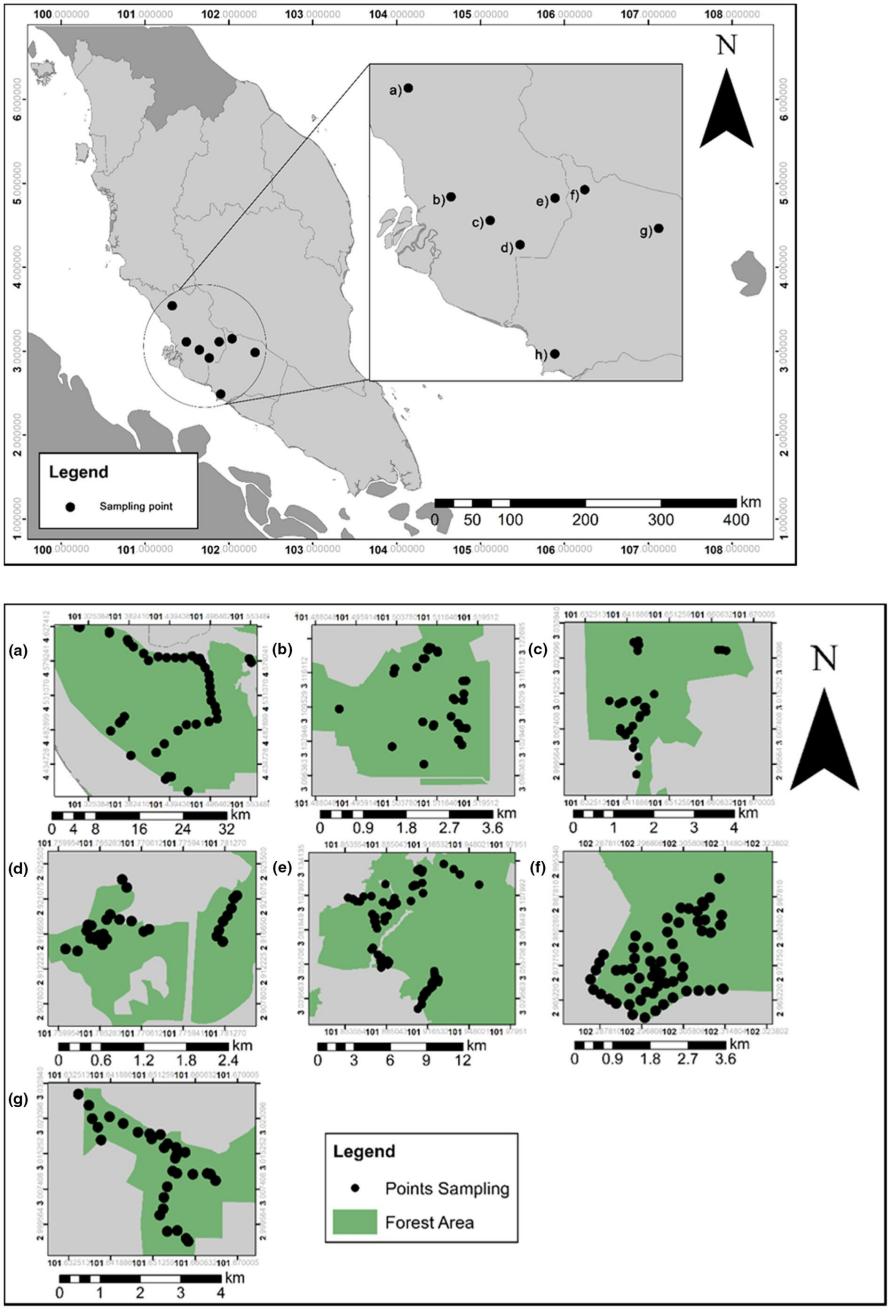
Map of study areas showing the sampling points in eight forest reserves in the states of Selangor and Negeri Sembilan, Peninsular Malaysia. The forest reserves were NSPSF (a), BCFR (b), AHFR (c), BFR (d), SLFR (e), KFR (f), PFR (g), and SMFR (h)

Our study area consisted of eight different forest reserves, which were located in the states of Negeri Sembilan and Selangor in Peninsular Malaysia (Table 1; Figure 1). Three forest reserves were located within Negeri Sembilan: Sungai Menyala Forest Reserve (SMFR) (2°29'39.61'' N, 101°53'22.27'' E), Kenaboi Forest Reserve (KFR) (3°7'39.72'' N, 102°2’56.4'' E), and Pasoh Forest Reserve (PFR) (2°33'58.95" N 102°11'56.76" E). Another five forest reserves were located in Selangor: North Selangor Peat Swamp Forest (NSPSF) (3°40'26.56'' N, 101°40'29.52'' E), Sungai Lalang Forest Reserve (SLFR) (3°3'26.31" N, 101°53'13.95" E), Ayer Hitam Forest Reserve (AHFR) (3°1'12.52" N, 101°38'46.76" E), Bangi Forest Reserve (BFR) (2°54'50.68" N, 101°46'1.18" E), and Bukit Cerakah Forest Reserve (BCFR) (3°6'34.43" N, 101°30'10.17" E). These eight forest reserves had different habitats that comprised unlogged and logged forest. The forest reserves were either fragmented or continuous forest landscapes. Forest reserves in Malaysia include both production forests subjected to logging and forests dedicated to conservation. KFR, NSPSF, SLFR, AHFR, BFR, and BCFR were selectively logged at least 30 years ago.

**TABLE 1:** Summary of camera trapping effort, site characteristics, and *Tragulus kanchil* images captured from eight forest reserves

Study area	Area (ha)	Forest type	Habitat type	Landscape type	No. sampling points	No. images of *T. kanchil* (mean ± SD)
North Selangor Peat Swamp Forest	78,000	Peat swamp	Logged forest	Continuous	45	1.13 ± 2.58
Sungai Lalang Forest Reserve	17,222	Lowland dipterocarp	Logged forest	Continuous	60	0.30 ± 1.51
Pasoh Forest Reserve	2450	Lowland dipterocarp	Unlogged forest	Continuous	60	0.15 ± 0.52
Kenaboi Forest Reserve	9420	Lowland dipterocarp	Logged forest	Continuous	30	0.07 ± 0.254
Bangi Forest Reserve	120	Lowland dipterocarp	Logged forest	Patch	30	1.83 ± 8.07
Ayer Hitam Forest Reserve	1200	Lowland dipterocarp	Logged forest	Patch	30	7.77 ± 16.78
Bukit Cerakah Forest Reserve	800	Lowland dipterocarp	Logged forest	Patch	30	7.63 ± 21.27
Sungai Menyala Forest Reserve	1280	Lowland dipterocarp	Unlogged forest	Patch	30	0.43 ± 1.01

### 2.2 Sampling design

Cameras were deployed at 315 sampling points using systematic sampling with a random starting point, where the first sampling point was chosen randomly within the forest reserves (Morrison et al., 2008). Each camera was deployed at least 200 m apart from another and at least 100 m from the trails used by humans. We selected the exact point of camera placement based on visible animal trails, footprints, animal scents, animal activity areas, and/or near streams (Sasidhran et al., 2016).

### 2.3 Camera trapping methods

**FIGURE 2:**
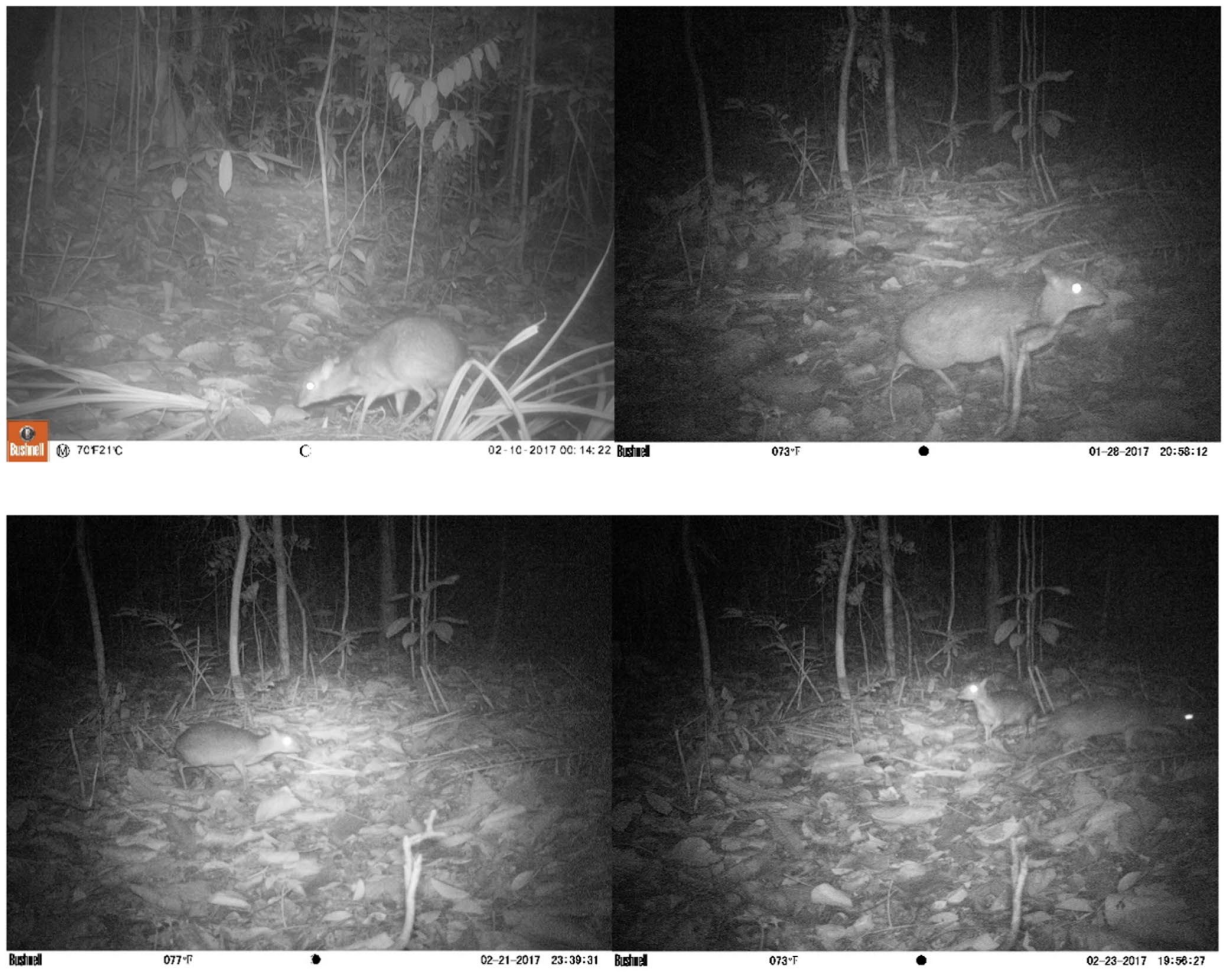
Images of *Tragulus kanchil* captured by camera traps in forest reserves

Camera trapping was conducted between March 2013 and April 2018, during the dry season. Thirty cameras (Bushnell Trophy Cam and Bushnell Trophy Cam HD) were used in the survey. The cameras operated 24 h per day and were left for 2 weeks or a month to maximize the number of detections and obtain sufficient data for analysis (Karanth & Nichols, 2002). In addition, the repeated theft of camera trap and limited site access in the field constrained sampling periods to 2 weeks or a month. The infrared feature of the Bushnell Trophy Cam consists of a sensor triggered by motion and heat. The camera was set to capture three images per second, with a 1‐ or 10‐s interval between exposures (i.e., taking three photographs per second or 10 s). The interval was set up randomly and varied between cameras throughout the study sites. The cameras were fixed on trees at the height of 30 cm to 50 cm above the ground at angles facing the animal trails. The images captured were sorted down to species level, with species other than *T. kanchil* excluded from the analysis (Figure 2). Overexposure and unclear images that led to unidentified species were also excluded (Sasidhran et al., 2016). Mouse‐deer detection in the camera traps was represented by the number of photographic images recorded at each of the 315 sites.

### 2.4 Assessment of site‐level and landscape‐level variables

To investigate the habitat quality requirements of *T. kanchil*, 12 habitat variables were measured and recorded in a vegetation plot (20 m × 20 m) that was established at each camera point. These variables included: (1) the number of saplings; (2) the number of trees with DBH between 5 cm and 45 cm; (3) the number of trees with DBH above 45 cm; (4) tree canopy cover (%); (5) the number of dead fallen trees; (6) the number of palms; (7) elevation (m), determined using Google Earth, which uses digital elevation model (DEM) data collected by NASA's Shuttle Radar Topography Mission (SRTM); (8) habitat type (logged or unlogged); (9) landscape type (continuous: >10,000 ha or connected to sizeable forested areas or patch: <10,000 ha and isolated); (10) type of forest (lowland dipterocarp forest or peat swamp forest); (11) sampling effort (two weeks or a month); and (12) distance from main road (km) (Table 2; Table S1‐S3). The selection of variables was based on previous studies (Jamhuri et al., 2018; Sasidhran et al., 2016; Tee et al., 2018) that were conducted to assess the mammal species present in a different type of tropical forest. We also used existing ecological information to guide us in selecting several site‐level and landscape variables (e.g., elevation, habitat type, type of forest). For example, some food plants favored by *T. kanchil* such as Sapium species are found in primary and secondary evergreen to deciduous rain forests, up to 800 m of altitude (Farida et al., 2006).

**TABLE 2:** Summary statistics for site‐level and landscape‐level variables in eight forest reserves. Unbalanced ANOVA was used to compare the site‐level and landscape‐level variables between forest reserves

Study area	No. saplings (mean ± SD)	No. trees with DBH between 5 cm and 45 cm (mean ± SD)	No. trees with DBH above 45 cm (mean ± SD)	Tree canopy cover (%) (mean ± SD)	No. dead fallen trees (mean ± SD)	No. palms (mean ± SD)	Elevation (m) (mean ± SD)	Distance from main road (km) (mean ± SD)
North Selangor Peat Swamp Forest	4.68 ± 5.04	5.01 ± 3.97	0.60 ± 0.79	64.00 ± 29.21	0.38 ± 0.49	2.05 ± 2.69	23.40 ± 10.15	2.98 ± 3.63
Sungai Lalang Forest Reserve	4.89 ± 2.02	12.44 ± 4.75	0.75 ± 0.56	91.20 ± 4.07	0.55 ± 0.54	1.51 ± 1.37	238.10 ± 80.26	1.26 ± 1.41
Pasoh Forest Reserve	3.96 ± 2.23	14.98 ± 7.32	1.29 ± 1.02	92.85 ± 4.33	0.47 ± 0.43	1.12 ± 1.26	132.10 ± 11.20	2.84 ± 0.82
Kenaboi Forest Reserve	47.67 ± 34.19	87.17 ± 12.51	0.90 ± 1.24	85.03 ± 12.00	2.13 ± 1.91	6.43 ± 7.20	309.50 ± 66.13	1.02 ± 0.90
Bangi Forest Reserve	27.60 ± 9.22	26.10 ± 9.92	0.53 ± 0.63	81.67 ± 12.34	1.37 ± 1.69	23.13 ± 19.37	74.03 ± 24.56	0.39 ± 0.23
Ayer Hitam Forest Reserve	26.13 ± 12.97	21.90 ± 8.13	0.33 ± 0.76	90.00 ± 11.45	0.83 ± 0.99	9.13 ± 7.97	73.90 ± 43.46	0.64 ± 0.47
Bukit Cerakah Forest Reserve	23.33 ± 18.12	23.93 ± 26.67	1.77 ± 4.55	94.67 ± 11.96	1.67 ± 1.63	11.00 ± 7.20	75.5 ± 39.75	0.35 ± 0.24
Sungai Menyala Forest Reserve	90.13 ± 51.41	6.30 ± 4.83	2.13 ± 1.70	83.40 ± 12.63	4.27 ± 2.70	5.33 ± 6.23	42.20 ± 13.71	1.55 ± 0.73
Variance ratio	71.67	192.09	4.71	21.43	32.82	33.34	183.54	17.55
*p* value	<.001	<.001	<.001	<.001	<.001	<.001	<.001	<.001

### 2.5 Data analysis

We used generalized linear mixed models (GLMMs) to determine the relationship between the occurrence of *T. kanchil* and the landscape variables. We developed two sets of models to prevent over‐fitting during model selection, one set including just site‐level variables, and another set including landscape variables. The GLMMs used a Poisson distribution and logarithm link function. We fixed the dispersion parameter for the variance of the response at 1 to adjust for overdispersion. We did not omit the data point with no detections, which we believe could compromise ecological explanations. Although our data seem to be zero‐inflated, that is, the number of zeros is so large that the data do not readily fit standard distributions, this does not necessarily mean a zero‐inflated model need to be used. This is because the explanatory variables would predict the zeros under a Poisson model.

We used *T. kanchil* detection in the camera traps, characterized by the number of photographic images captured at each of the 315 sampling points as a proxy for the occurrence. The occurrence of *T. kanchil* was used as response variable, which is a function of 12 explanatory variables in the candidate models. To control for correlated structure in the data, the location of the camera trapping point (i.e., forest reserve), year, and time lapse between exposures were included as the random effect. Correlation tests were performed for multicollinearity among the variables in the global models that included landscape variables and in situ covariates. No variable had correlation higher than 0.7 and hence all explanatory variables were included in the analysis (Dormann et al., 2013).

To perform model selection, we fitted all possible regression models and evaluated these according to an Information Theoretic Approach. In this way, a number of best regression models were selected using computer‐intensive statistical model building process. We used Akaike's Information Criterion (AIC) to determine the most parsimonious model based on the minimum values of AIC and calculate the AIC weights (Burnham & Anderson, 2002). We reported adjusted *R* ^2^ values for every model to complement the AIC values. The candidate models from all possible combinations of parameters were selected and fitted to the data and ranked by ΔAIC values (AIC−AIC_min_). The statistical analysis was conducted using GenStat 12th version (VSN International, Hemel Hempstead, UK).

## 3 RESULTS

### 3.1 General patterns of *T. kanchil* distribution

Out of 5140 images, 610 images of *T. kanchil* were recorded at 56 sampling points. BCFR had the highest number of *T. kanchil* detections (229 images), followed by AHFR with 233 images, BFR with 55 images, NSPSF with 51 images, SLFR with 18 images, SMFR with 13 images, PFR with 9 images, and KFR with only 2 images. The number of *T. kanchil* detections varied across reserves (Table 1). We recorded melanistic leopard (*Panthera pardus*) that potentially preys on *T. kanchil* only in NSPSF, but none from other forest reserves. Mesopredators such as dhole (*Cuon alpinus*) and clouded leopard (*Neofelis nebulosa*) were not recorded.

### 3.2 Drivers of *T. kanchil* occurrence

**FIGURE 3:**
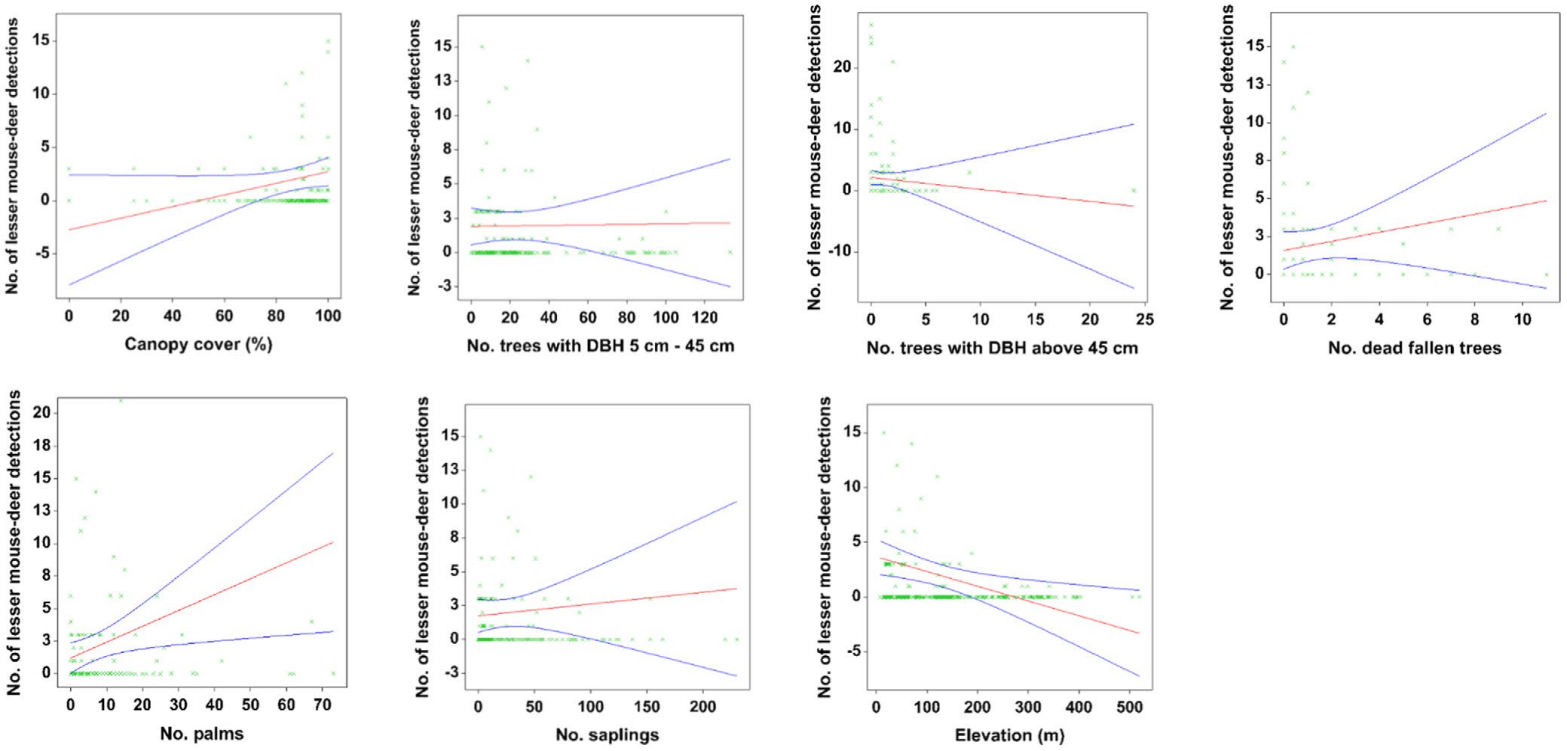
Scatterplots with 95% confidence intervals (blue) on the regression (red) line showing the relationships between the detection of *Tragulus kanchil* and site‐level variables

Out of 13 explanatory variables, 11 variables were strongly correlated with the detection of *T. kanchil*. The most parsimonious site‐level model explained 36.74% of the variation in lesser mouse‐deer occurrence corresponded to the best subsets with eight terms (Table 3). The model accounted for 50% of the Akaike weights in the model set. The detection of *T. kanchil* increased with the percentage of canopy cover, the number of trees with DBH of 5 cm to 45 cm, the number of saplings, the number of palms, and the number of dead fallen trees (Table 4; Figure 3). In contrast, the detection of *T. kanchil* decreased with the number of trees with DBH above 45 cm and elevation. The detection of *T. kanchil* was not affected by the sampling effort (Table 4; Figure 3).

**TABLE 3:** Best subsets from the selected models, with the most parsimonious model with comparatively high adjusted *R* ^2^ and the lowest value of AIC in bold

Model	Explanatory variables	Adjusted *R* ^2^	AIC	∆*_i_*	Relative likelihoods	Akaike weights
Site‐level	Elevation + (1|Location + Year + Time lapse)	14.50	2758.2	743.3	3.9306 × 10^–162^	1.9652 × 10^–162^
	Elevation + Canopy cover+ (1|Location + Year + Time lapse)	26.73	2358.8	343.9	2.10409 × 10^–75^	1.05201 × 10^–75^
	Elevation + Canopy cover + Sampling effort+ (1|Location + Year + Time lapse)	28.85	2285.4	270.5	1.82672 × 10^–59^	9.13332 × 10^–59^
	Elevation + Canopy cover + Abundance of trees with DBH 5–45 cm + Abundance of trees with DBH >45 cm + (1|Location + Year + Time lapse)	35.27	2075.4	60.5	7.28772 × 10^–14^	3.64375 × 10^–14^
	Elevation + Canopy cover + Abundance of trees with DBH 5–45 cm + Abundance of trees with DBH >45 cm + Dead fallen tree abundance + (1|Location + Year + Time lapse)	36.12	2043.7	28.8	5.5739 × 10^–7^	2.78687 × 10^–7^
	Elevation + Canopy cover + Abundance of trees with DBH 5–45 cm + Abundance of trees with DBH >45 cm + Dead fallen tree abundance + Sapling abundance + (1|Location + Year + Time lapse)	36.80	2017.6	2.7	0.2592	0.1296
	**Elevation + Canopy cover + Abundance of trees with DBH 5–45 cm + Abundance of trees with DBH >45 cm + Dead fallen tree abundance + Sapling abundance + Palm abundance + (1|Location + Year + Time lapse)**	**36.74**	**2014.9**	**0**	**1**	**0.5000**
	Elevation + Canopy cover + Abundance of trees with DBH 5–45 cm + Abundance of trees with DBH >45 cm + Dead fallen tree abundance + Sapling abundance + Palm abundance + Sampling effort+ (1|Location + Year + Time lapse)	36.58	2015.5	0.6	0.740818221	0.3704
Landscape‐level	Landscape type + (1|Location + Year + Time lapse)	19.10	2610.1	259.4	4.699 × 10^–57^	3.34648 × 10^–57^
	Landscape type + Habitat type + (1|Location + Year + Time lapse)	25.72	2391.2	40.5	1.6052 × 10^–9^	1.1432 × 10^–9^
	Landscape type + Habitat type + Forest type + (1|Location + Year + Time lapse)	26.56	2358.8	8.1	0.0174	0.0124
	**Landscape type + Habitat type + Forest type + Sampling effort + (1|Location + Year + Time lapse)**	**26.64**	**2350.7**	**0**	**1**	**0.7122**
	Landscape type + Habitat type + Forest type + Distance from nearest road + Sampling effort + (1|Location + Year + Time lapse)	26.40	2352.6	1.9	0.3867	0.2754

**TABLE 4:** Coefficient of important site‐level and landscape‐level variables

Variable	Coefficient	SE
Canopy cover	0.033	0.004
No. tree with DBH 5 cm–45 cm	0.022	0.004
No. tree with DBH above 45 cm	−0.163	0.032
No. dead fallen trees	0.337	0.032
No. palms	0.020	0.004
No. saplings	0.001	0.002
Elevation	−0.028	0.002
Landscape type		
Continuous forest	0.000	1.197^a^
Patch	4.113	
Habitat type		
Logged forest	0.000	0.793^a^
Unlogged forest	−1.719	
Forest type		
Lowland dipterocarp	0.000	2.484^a^
Peat swamp	2.855	
Sampling effort		
1 month	0.000	1.326^a^
2 weeks	−2.009	

^a^ Standard error of differences.

At landscape level, the most parsimonious model had an adjusted *R* ^2^ of 26.64% and included four terms (Table 3). The model accounted for 71.22% of the Akaike weights in the model set. A high frequency of camera‐trap images showed two logged lowland dipterocarp forest patches which had very high detection rates—some 4–6 times higher than any other sites. Our result showed that forest patches had the detection of *T. kanchil* 4.113 times greater than continuous forests (Table 4). The detection of *T. kanchil* was 2.855 times higher in peat swamp forest compared to lowland dipterocarp landscapes; however, confidence in the coefficient estimate was low and overlapped zero (Table 4). Surprisingly, the detection of *T. kanchil* was 1.7193 times lower in the unlogged forests compared to the logged forests (Table 4). The distance from nearest roads did not affect the detection of *T. kanchil*.

## 4 DISCUSSION

### 4.1 Distribution patterns

The logged forest reserves (BFR, AHFR, BCFR, NSPSF, and SLFR) had higher *T. kanchil* detection rates compared to the unlogged forest reserves (SMFR, KFR, PFR, and KFR). These results support previous findings, which stated that chevrotains (*Tragulus* spp.) were relatively more common in the logged forest than in unlogged forest (Granados et al., 2016). A study in Sabah concludes unlogged forest is the preferred habitat by *T. kanchil* (Heydon & Bulloh, 1997), but this present study found the opposite because of two possible factors. First, the forest reserves we surveyed were selectively logged at least 30 years ago, whereas Heydon and Bulloh (1997) surveyed forests in Sabah that were logged after 2, 5, and 12 years. Second, they used line‐transect surveying, whereas we deployed camera trap, which is more successful at detecting elusive species in tropical forests than line transects (Espartosa et al., 2011; Silveira et al., 2003), and we therefore had more confidence in our result. Moreover, throughout sampling in the field across all eight forests for nearly 6 years, only once we had directly encountered a mouse‐deer in the SLFR (a continuous forest but already logged).

### 4.2 Site‐level and landscape‐level variable preferences

The detection of *T. kanchil* across all study sites was highly variable, especially with respect to forest, habitat, and landscape types. Out of eight forest reserves, six were selectively logged at least 30 years ago. This may contribute to the habitat heterogeneity and complexity in the reserves. Tropical forests are heterogeneous and patchy, even without strong anthropogenic disturbances (Whitmore, 1998). Canopy gaps occur in both undisturbed and disturbed forests as gaps are caused by the death of one or more trees in tropical ecosystems (Kadmon, 2001). Perhaps there were more large gaps in logged forests whereas more small gaps in unlogged forests. Resprouting has been found to be more prevalent underneath small canopy gaps than in large ones (Brown, 2004).

After 30 years or more, through plant succession, tree canopy in logged forests could regenerate and may result in lower light intensity. Our results showed that the *T. kanchil* detection increased with the percentage of canopy cover. This suggests that *T. kanchil* prefers habitats of dense evergreen closed‐canopy forest. However, the creation of a small canopy gap may increase solar radiation reaching the forest floor and promote the growth of seedlings including those edible to *T. kanchil* through the enhanced light levels found in the gap (Brown, 2004; Burslem, 2004). Otherwise, only the most shade‐tolerant plant species can survive and grow in the deep shade of a forest understory (Brown, 2004). In addition, Matsubayashi et al. (2003) found in Borneo that lesser mouse‐deer preferred dense undergrowth of creeping bamboo (*Dinochloa* spp.) with canopy gaps, which is similar to the BCFR and AHFR which included forest areas with lots of bamboo vegetation that resulted in most detection of mouse‐deer.

We also found that *T. kanchil* detection increased with the abundance of trees with a DBH of 5 cm to 45 cm and decreased when tree DBH was greater than 45 cm. These results were supported by previous research, which showed that small ungulates were very active and moved long distances mostly in crown gap areas with dense undergrowth which provide shelter during the day (Matsubayashi et al., 2003). These habitat characteristics are also suitable for foraging as this species predominantly consumes fallen fruits and young leaves from pioneer plants (Bodmer, 1990; Prasad et al., 2010).

In addition, our results revealed that *T. kanchil* detections increased with a high number of dead fallen trees. This was similar to a study in Borneo forest that showed *T. kanchil* rested under shelters such as dead fallen trees or branches. However, it was commonly found foraging in more dense forests (Matsubayashi et al., 2003). The other covariates also support *T. kanchil's* preference for areas that are associated with forest gaps. For example, the detection of *T. kanchil* increased in areas with a high number of saplings. *Tragulus kanchil* possibly relies on food resources close to the dense forest floor in open canopy areas such as short vegetation and fallen fruits (Jayasekara et al., 2007; Matsubayashi et al., 2003). Areas with understory cover and high leaf litter are suitable for *T. kanchil* to forage for food and provide refuge for small‐bodied ungulates.

Our study also showed that the detection of *T. kanchil* increased with the number of palms. Palms can coexist with the shrub plants, which is an essential food resource for *T. kanchil* (Farida et al., 2006; Matsubayashi et al., 2003). *Tragulus kanchil* is partly frugivorous species that are heavily dependent on fallen fruit for nourishment, browsing fruit from pioneer tree species (Heydon & Bulloh, 1997; Meijaard & Sheil, 2008). Matsubayashi et al. (2003) suggested that they are active in open canopy areas characterized by dense undergrowth plants in logged forests. It has been reported that mouse‐deer feeds on 50 wild plant species consisting of 22 families (Farida et al., 2006).

Our study suggested that the detections of *T. kanchil* were associated with the type of forest. Detection was lower in the lowland dipterocarp forest compared to the peat swamp forest. Our research also found that *T. kanchil* was more likely to be detected in lower elevation forests such as NSPSF. Peat swamp forest provides food resources, dense vegetation cover, which is suitable for hiding, foraging, and is commonly the most frequented place where *T. kanchil* can be found throughout the year (Matsubayashi et al., 2003; Ramesh et al., 2013; Sasidhran et al., 2016).

### 4.3 Impact of human disturbances

Our results indicated that the occurrence of *T. kanchil* was greater in patches than continuous forests. This finding showed that *T. kanchil* populations could persist in forest patches. This particular finding can be explained by the absence of natural predators in the forest patches (Khalidah et al., 2021; Tee et al., 2018). *Tragulus kanchil* was probably preyed on by leopards, feral dogs, and pythons in the forest reserves. In addition, *T. kanchil* might thrive in forest patches because of the lack of competition and high resource availability (e.g., fruits of pioneer species).

*Tragulus kanchil* was also positively impacted by human disturbance. The mouse‐deer was more likely to inhabit the logged forest compared to unlogged forest. This is because the logged forest may provide more plant food resources in their understory. Unlike larger grazing and browsing species, *T. kanchil* tends to be a selective feeder and it does not need to gather large quantities of food daily (Heydon & Bulloh, 1997). *Tragulus kanchil* spends more time selecting more edible leaves, shoots, flowers, and fruits (Matsubayashi & Sukor, 2005). In contrast, Magintan et al. (2017) suggest that animal abundance in the unlogged forest was higher than the logged forests most likely due to the abundance of food plants (e.g., *Sapium baccatum*) eaten by *T. kanchil*.

*Tragulus kanchil* can be found in disturbed and fragmented areas (e.g., plantations, rural areas, and degraded forest) across Malaysia (Jambari et al., 2019; Magintan et al., 2017; Ramesh et al., 2013; Tee et al., 2018). In continuous forest such as KFR, *T. kanchil* was not detected at all. This may be likely linked to hunting pressure by the aboriginal people who reside nearby the forest reserve. At PFR, all large‐bodied mammals, except wild pigs, were decimated due to hunting activities over a similar period (Ickes & Thomas, 2003).

The mouse‐deer populations have been threatened by extensive land clearing and poaching across their known habitat (Azhar et al., 2013; Petersen et al., 2020). Nguyen et al. (2019) suggest that snares laid by hunters have pushed the species to the brink of extinction in Vietnam. However, we did not encounter any traps in our study area. Poaching is believed to occur year‐round although hunting is prohibited within the forest reserves (Goldthorpe & Neo, 2011).

## 5 CONCLUSION

The results from this study provide valuable information to stakeholders supporting the conservation of existing forest patches irrespective of size. To conserve the habitat of *T. kanchil* in the forest reserves, they should monitor and manage site‐level habitat quality. The occurrence of *T. kanchil* was influenced by forest fragmentation. However, both forest patch and continuous forest are equally crucial for conserving *T. kanchil* populations. Our data give a preliminary indication that *T. kanchil* may prefer peat swamps forests, which justifies the conservation of peat swamp forests as one of the critical habitats in Southeast Asia. This study also showed that logged forest had a higher detection of *T. kanchil* compared to the unlogged forest. This suggests that logged forest should not be sidelined because of its conservation value for *T. kanchil*. We suggest more research into the anthropogenic threats in elsewhere across Southeast Asia where *T. kanchil* lives to protect them better. *Tragulus kanchil* has a good chance of survival in forestry landscapes if the key threats are removed.

## CONFLICT OF INTEREST

The authors declare that they have no conflict of interest.

## AUTHOR CONTRIBUTIONS

**Muhammad Hazwan:** Conceptualization (equal); Data curation (equal); Formal analysis (equal); Investigation (equal); Methodology (equal); Validation (equal); Visualization (equal); Writing – original draft (equal). **Liza D. Samantha:** Data curation (equal); Investigation (equal); Methodology (equal); Validation (equal); Writing – review & editing (equal). **Sze Ling Tee:** Investigation (equal); Methodology (equal); Validation (equal); Writing – review & editing (equal). **Norizah Kamarudin:** Conceptualization (equal); Investigation (equal); Methodology (equal); Project administration (equal); Supervision (equal). **Ahmad R. Norhisham:** Supervision (equal); Writing – review & editing (equal). **Alex M. Lechner:** Writing – review & editing (equal). **Badrul Azhar:** Conceptualization (equal); Data curation (equal); Formal analysis (equal); Investigation (equal); Methodology (equal); Project administration (equal); Resources (equal); Software (equal); Supervision (equal); Visualization (equal); Writing – review & editing (equal).

## Supporting information

Table S1‐S3

## Data Availability

Empirical data have been archived in Data Dryad: https://doi.org/10.5061/dryad.jdfn2z38s.
