# Supplementary material for: Habitat fragmentation and logging affect the occurrence of lesser mouse‐deer in tropical forest reserves
Source: Ecol Evol. 2022 Mar 18;12(3):e8745. doi: 10.1002/ece3.8745 (PMC8933326; doi:10.1002/ece3.8745)
Supplement: Supplementary file 1 — Table S1‐S3 [file ECE3-12-e8745-s001.docx]

**Supplementary information**

**Table S1**. Summary statistics for site-level and landscape-level variables in different habitat types. Unbalanced ANOVA was used to compare the site-level and landscape-level variables between logged forest and unlogged forest.

| **Habitat type** | **No. saplings (mean ± SD)** | **No. trees with DBH between 5 cm to 45 cm (mean ± SD)** | **No. trees with DBH above 45 cm (mean ± SD)** | **Tree canopy cover (%) (mean ± SD)** | **No. dead fallen trees (mean ± SD)** | **No. palms (mean ± SD)** | **Elevation (m) (mean ± SD)** | **Distance from main road (km) (mean ± SD)** |
| --- | --- | --- | --- | --- | --- | --- | --- | --- |
| Logged forest | 18.87 ± 21.37 | 25.53 ± 28.00 | 0.79 ± 1.84 | 83.97 ± 19.00 | 1.02 ± 1.35 | 7.44 ± 11.07 | 139.20 ± 117.00 | 1.25 ± 2.03 |
| Unlogged forest | 32.68 ± 50.33 | 12.09 ± 7.75 | 1.57 ± 1.34 | 89.70 ± 9.19 | 1.74 ± 2.40 | 2.52 ± 4.20 | 102.10 ± 44.27 | 2.41 ± 1.00 |
| Variance ratio | 11.72 | 20.10 | 13.49 | 7.49 | 11.23 | 16.75 | 8.55 | 26.51 |
| p value | <0.001 | <0.001 | <0.001 | 0.007 | <0.001 | <0.001 | 0.004 | <0.001 |

**Table S2**. Summary statistics for site-level and landscape-level variables in different landscape type. Unbalanced ANOVA was used to compare the site-level and landscape-level variables between continuous forest and patch.

| **Landscape type** | **No. saplings (mean ± SD)** | **No. trees with DBH between 5 cm to 45 cm (mean ± SD)** | **No. trees with DBH above 45 cm (mean ± SD)** | **Tree canopy cover (%) (mean ± SD)** | **No. dead fallen trees (mean ± SD)** | **No. palms (mean ± SD)** | **Elevation (m) (mean ± SD)** | **Distance from main road (km) (mean ± SD)** |
| --- | --- | --- | --- | --- | --- | --- | --- | --- |
| Continuous forest | 11.14 ± 20.67 | 23.00 ± 28.57 | 0.90 ± 0.93 | 84.48 ± 18.94 | 0.73 ± 1.05 | 2.27 ± 3.70 | 166.90 ± 112.00 | 2.11 ± 2.16 |
| Patch | 41.80 ± 39.67 | 19.56 ± 16.75 | 1.19 ± 2.56 | 87.43 ± 13.04 | 2.03 ± 2.26 | 12.15 ± 13.18 | 66.41 ± 35.15 | 0.73 ± 0.67 |
| Variance ratio | 80.93 | 1.44 | 2.03 | 2.25 | 47.89 | 97.18 | 91.07 | 45.90 |
| p value | <0.001 | 0.231 | 0.155 | 0.134 | <0.001 | <0.001 | <0.001 | <0.001 |

**Table S3**. Summary statistics for site-level and landscape-level variables in different forest types. Unbalanced ANOVA was used to compare the site-level and landscape-level variables between lowland dipterocarp forest and peat swamp forest.

| **Forest type** | **No. saplings (mean ± SD)** | **No. trees with DBH between 5 cm to 45 cm (mean ± SD)** | **No. trees with DBH above 45 cm (mean ± SD)** | **Tree canopy cover (%) (mean ± SD)** | **No. dead fallen trees (mean ± SD)** | **No. palms (mean ± SD)** | **Elevation (m) (mean ± SD)** | **Distance from main road (km) (mean ± SD)** |
| --- | --- | --- | --- | --- | --- | --- | --- | --- |
| Lowland dipterocarp forest | 25.84 ± 34.58 | 24.47 ± 25.67 | 1.08 ± 1.85 | 89.21 ± 10.28 | 1.37 ± 1.83 | 6.70 ± 10.46 | 146.20 ± 101.00 | 1.35 ± 1.24 |
| Peat swamp forest | 4.68 ± 5.04 | 5.01 ± 3.97 | 0.60 ± 0.79 | 64.00 ± 29.21 | 0.38 ± 0.49 | 2.05 ± 2.69 | 23.40 ± 10.15 | 2.98 ± 3.63 |
| Variance ratio | 16.74 | 25.68 | 3.03 | 116.31 | 12.83 | 8.76 | 66.20 | 32.50 |
| p value | <0.001 | <0.001 | 0.083 | <0.001 | <0.001 | 0.003 | <0.001 | <0.001 |
